# Automatic Inter-Frame Patient Motion Correction for Dynamic Cardiac PET Using Deep Learning

**DOI:** 10.1109/TMI.2021.3082578

**Published:** 2021-11-30

**Authors:** Luyao Shi, Yihuan Lu, Nicha Dvornek, Christopher A. Weyman, Edward J. Miller, Albert J. Sinusas, Chi Liu

**Affiliations:** Department of Biomedical Engineering, Yale University, New Haven, CT 06512 USA; Department of Radiology and Biomedical Imaging, Yale University, New Haven, CT 06512 USA; Department of Radiology and Biomedical Imaging, Yale University, New Haven, CT 06512 USA; Department of Internal Medicine (Cardiology), Yale University, New Haven, CT 06512 USA; Department of Internal Medicine (Cardiology), Yale University, New Haven, CT 06512 USA; Department of Internal Medicine (Cardiology), Yale University, New Haven, CT 06512 USA; Department of Biomedical Engineering and the Department of Radiology and Biomedical Imaging, Yale University, New Haven, CT 06512 USA

**Keywords:** PET, myocardial perfusion, motion correction, deep learning

## Abstract

Patient motion during dynamic PET imaging can induce errors in myocardial blood flow (MBF) estimation. Motion correction for dynamic cardiac PET is challenging because the rapid tracer kinetics of 82Rb leads to substantial tracer distribution change across different dynamic frames over time, which can cause difficulties for image registration-based motion correction, particularly for early dynamic frames. In this paper, we developed an automatic deep learning-based motion correction (DeepMC) method for dynamic cardiac PET. In this study we focused on the detection and correction of inter-frame rigid translational motion caused by voluntary body movement and pattern change of respiratory motion. A bidirectional-3D LSTM network was developed to fully utilize both local and nonlocal temporal information in the 4D dynamic image data for motion detection. The network was trained and evaluated over motion-free patient scans with simulated motion so that the motion ground-truths are available, where one million samples based on 65 patient scans were used in training, and 600 samples based on 20 patient scans were used in evaluation. The proposed method was also evaluated using additional 10 patient datasets with real motion. We demonstrated that the proposed DeepMC obtained superior performance compared to conventional registration-based methods and other convolutional neural networks (CNN), in terms of motion estimation and MBF quantification accuracy. Once trained, DeepMC is much faster than the registration-based methods and can be easily integrated into the clinical workflow. In the future work, additional investigation is needed to evaluate this approach in a clinical context with realistic patient motion.

## I. Introduction

POSITRON emission tomography (PET) myocardial perfusion imaging has been shown to improve the diagnostic accuracy of coronary artery disease (CAD) as compared to other non-invasive imaging modalities [1]. Absolute quantification of myocardial blood flow (MBF) and myocardial flow reserve (MFR) using dynamic PET has shown superior diagnostic and prognostic value as compared to the conventional relative myocardial perfusion imaging [2]. In typical dynamic PET, a dynamic sequence of images is acquired over several minutes, starting from the injection of radio-labeled tracer until myocardium is well perfused. Regions of interest are usually defined on the reconstructed dynamic images to sample the time-activity curves (TAC) in the myocardium tissue and left ventricle (LV) cavity. These TACs can be further processed via kinetic modeling to quantify MBF.

Patient motion during dynamic imaging, which typically includes respiratory motion, cardiac motion and voluntary body motion, can induce errors in MBF estimation [3], [4]. Specifically, patient motion can present as inter-frame motion, which can cause misalignment of the heart between successive dynamic image frames and result in distorted TACs due to inconsistent ROI sampling. On the other hand, intra-frame motion can lead to blurred images as well as inaccurate image-derived input function (IDIF) measured within LV cavity. Additional errors could also be introduced by the mismatch between PET and CT-based attenuation map caused by patient motion [5].

Respiratory and cardiac motion correction methods in PET have been investigated in the past [6], [7]. External motion tracking markers or sensors have been used in some studies [5], [8] to track respiratory motion at a high temporal resolution. Electrocardiography (ECG) is still the gold-standard when it comes to track the cardiac motion due to high temporal resolution. However, such tracking systems typically require extra setup time and may not always be accessible. Alternatively, data-driven motion detection methods [9]-[11] without the need of external devices are preferred to facilitate easier clinical translation of motion correction. Nonetheless, few studies focused on the correction of inter-frame motion for ^82^Rb cardiac dynamic PET imaging due to its challenging nature. The rapidly changing biodistribution of ^82^Rb leads to dynamic changes in the distribution of the radiotracer from frame to frame over time, which can cause difficulties for image registrations. The accuracy of image registration typically relies on the similarity between the two images to be registered with each other. Due to the rapid tracer kinetics, the tracer distribution in one dynamic frame can be substantially different from another frame, which can result in inaccurate or even failed image registration. This is particularly the case for early dynamic frames (blood pool phase), and therefore existing motion-correction studies [12]-[14] have largely focused on the later dynamic frames (myocardial tissue frames). To address this challenge, Hunter *et al*. [15] proposed to correct patient body motion for dynamic cardiac PET-CT by attenuation-emission alignment according to projection consistency conditions. The method performed equally well on both early and later frames, but was only evaluated on simulation and phantom data. Lee *et al*. [16] developed an automated motion correction framework for dynamic ^82^Rb cardiac PET. The right-ventricle blood pool (RVBP) phase, left-ventricle blood pool (LVBP) phase and tissue-phase were first identified using an automated algorithm, then each dynamic frame was rigidly aligned to match a fixed later tissue-phase summed frame to achieve motion correction. Normalized gradient fields, instead of image intensities, were used in registration to account for rapid tracer kinetics during the blood pool phase. Lee’s results were in good agreement with the manual motion correction results in the evaluation of clinical studies, and the method applies to the entire dynamic sequence. However, Lee’s method’s performance in the transition frames (in between LV blood phase and tissue phase) is questionable due to the activity being in both blood pool and myocardial tissue, causing unidentifiable boundaries and therefore unreliable gradient to compute registration. The RV frames were also not validated due to a lack of manual motion correction. Since the tissue-phase frames were summed to provide a reference image, the reference image might be blurred due to the interframe motion and could lead to inaccurate motion estimation.

Deep learning has demonstrated its promising performance in many medical imaging tasks, including image enhancement [17]-[20], image registration [21], image segmentation [22], [23], image generation [24]-[26], and computer-aided diagnosis [27]. However, using deep learning for motion detection on sequential images has been mostly unexplored. Recently, recurrent neural network (RNN) and long short-term memory (LSTM) [28], [29] have achieved great success in processing sequential multimedia data and yielded state-of-the-art results in video and signal processing [30], [31]. Li *et al*. [32] also obtained promising results by applying convolutional LSTM on X-ray fluoroscopic images to recover cardiac and respiratory signal, although their study was focused on extracting 1D motion signal from a sequence of 2D images with similar anatomical structures. Guo *et al*. [33] used LSTM to classify respiratory signals into regular and irregular breathing patterns to guide optimal motion correction strategies in PET.

In this paper, we developed automatic motion correction for dynamic cardiac PET using deep learning (DeepMC) for the first time, to the best of our knowledge. In this study, we focus on detection and correction of the inter-frame rigid translational motion caused by body motion and change in the pattern of respiratory motion. The intra-frame motion resulting from cardiac and respiratory motion are averaged within the frames and are not considered in this study. A bidirectional-LSTM [34] network structure was employed to utilize both local and nonlocal temporal information in the 4D dynamic image data for motion detection. The proposed method was further evaluated on patient data with both simulated and real motion, in terms of motion estimation and MBF quantification accuracy.

## II. Materials and Methods

### A. Dataset

A total of 160 anonymized clinical ^82^Rb PET rest and regadenoson-induced stress studies were included from Yale New Haven Hospital from December 2019 to January 2020. The PET data were acquired using a Discovery 690 PET/CT scanner (GE Healthcare, Waukesha, WI). The data anonymization for this research was approved by the Institutional Review Board of Yale University. ^82^Rb was delivered via a programed infusion using a commercial ^82^Rb generator (Bracco Diagnostics Inc.) with a weight-based targeted injection dose of 20-35 mCi depending on patient body mass index. The total acquisition time for each scan was 7 min, but only the first 6 min 10 s data were included in the rebinning process according to the clinical setting. Listmode data were rebinned into dynamic image sequence of 14 × 5s, 6 × 10s, 3 × 20s, 3 × 30s, and 1 × 90s timeframes. Images were reconstructed using OSEM with 2 iterations and 24 subsets, resulting in 128 × 128 × 47 voxels of size 3.125 × 3.125 × 3.270 mm. Images were filtered with Butterworth filter with a cutoff frequency of 21 mm^−1^ and an order of 5. Corrections for isotope decay, photon attenuation and scatter, random and prompt-gamma coincidences, detector efficiency, and deadtime were all applied to reconstruct quantitative images of activity concentration (Bq/mL), according to our standard clinical practice. Note that each dynamic frame was reconstructed independently. Therefore, the scatter estimation was derived from each individual frame’s emission data, instead of from an initial static reconstruction. CT and ^82^Rb PET image were manually registered using the vendor ACQC package.

With the Corridor 4DM software that was used in our clinical setting, one of the technologists performed motion correction frame by frame manually until no visual motion can be observed between frames. The manual motion correction results were then double checked by one of the research group members. Then, the 4DM software can automatically display the motion magnitudes resulted from the manual motion correction. A total of 85 motion-free scans (55 rest and 30 stress scans) from 59 patients were identified from the 160 patients’ studies. The motion-free scans were defined as scans with a total motion (in all the frames) no more than 3 mm, same as the convention used in [4]. Motion was defined as the translational shift of any frame compared with the last frame (frame 27 with the longest duration). Rotational and non-rigid motions were not considered in this study, because in clinical practice the majority of manual corrections were done for translational motion. The 85 motion-free scans were later divided into two subsets, with 65 used for training and 20 used for evaluation of the network with added simulated motion. Additionally, 10 studies (4 rest and 6 stress scans) from 9 patients that have at least 5 frames with mild or severe motion (larger than 3 mm) [4] were selected for testing the network. A summary of the characteristics of the included scans were given in Table I.

### B. Motion Simulation

**Fig. 1.**
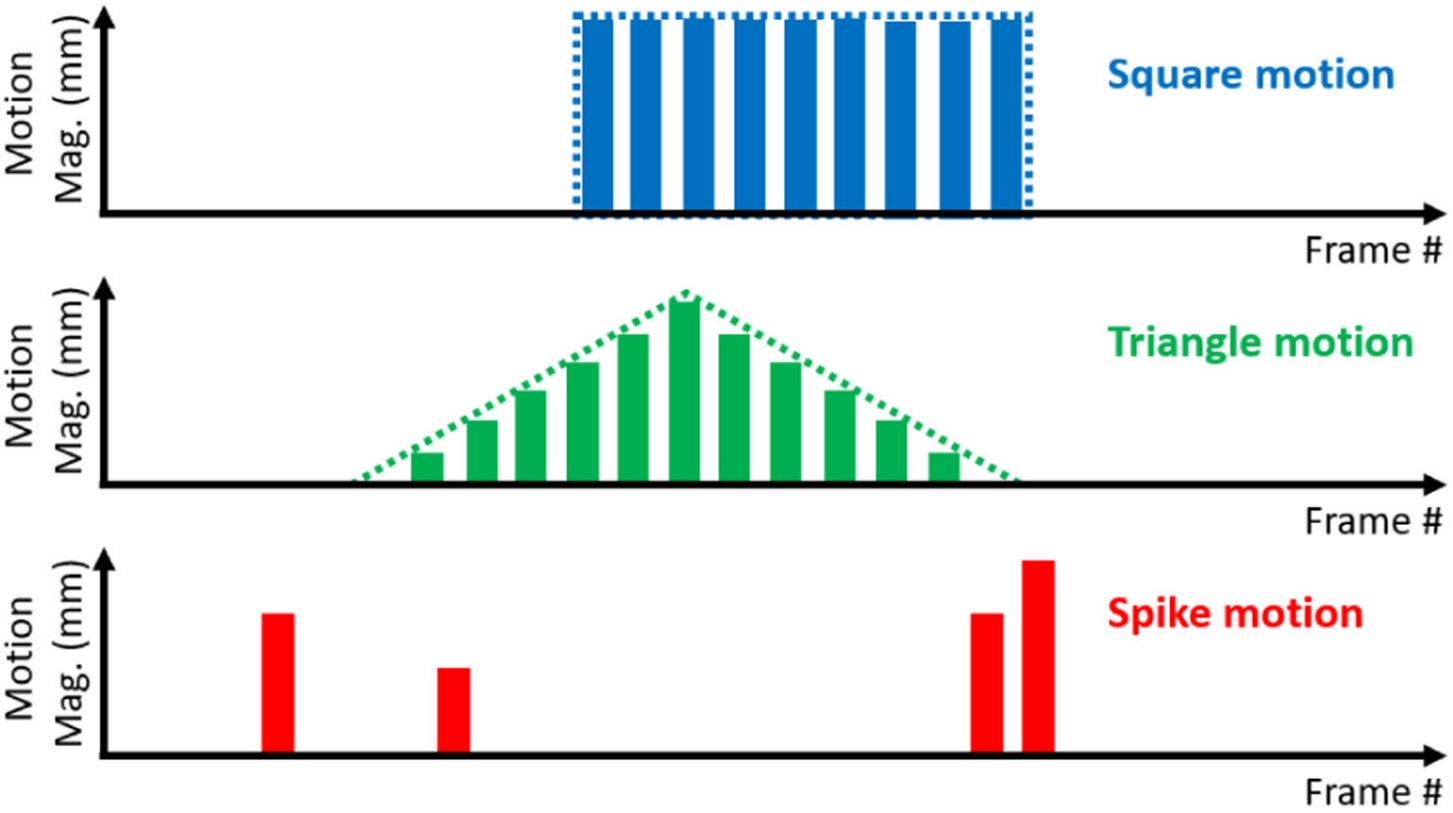
An illustration of the three types of motion in simulation for network training. This illustration only shows 1-D motion displacement patterns with the motion magnitudes changing over frames, whereas the motion directions are not reflected in this figure.

Three types of motion were simulated during training of the network, namely “square motion”, “triangle motion” and “spike motion”. Square motion simulates a type of patient’s motion that is consistent across multiple frames with the same magnitude and direction in the 3D Euclidian space, triangle motion refers to the scenario where patient motion gradually builds up along one direction and then moves back to the original position across several frames, and spike motion simulates random motion, where the motion magnitude and direction in each frame can be independent. An illustration of the three types of motion is shown in Fig. 1. We believe the combination of three basic motion types can approximately represent the realistic motion patterns for training purpose, even though the realistic motion could be more complicated.

For each dynamic image sequence, a window of 64 × 64 × 36 centered around the mid-point of the septal wall (manually identified currently) was cropped from the last image frame (frame 27). Motion in any other frame was simulated by shifting the cropping window in the Euclidian space, with a step size of 0.1 pixel (using linear interpolation) along each axis.

### C. Network Architectures

To predict the motion of a given frame, not only the adjacent frames can provide local information in the form of relative motion, the non-adjacent frames can also provide nonlocal information about the cross-frame motion correlation due to inertia, especially for the motion with long-duration that affects multiple frames (e.g., the previously mentioned triangle motion and spike motion). In addition, knowing the spatial tracer distribution change in time can also potentially help motion detection. However, this also requires the network to “see” multiple frames simultaneously. Recurrent neural network approaches such as long short-term memory (LSTM) [29] are capable of interpreting and summarizing patterns among correlated data samples, which is an ideal network architecture for our problem. The cell state of LSTM allows nonlocal information from non-adjacent frames to be transferred all the way along the sequence, to assist motion prediction for each individual frame. In this work we used a bidirectional 3D convolutional LSTM (convLSTM3D) network to allow information to flow along both ways between the early and late phases.

**Fig. 2.**
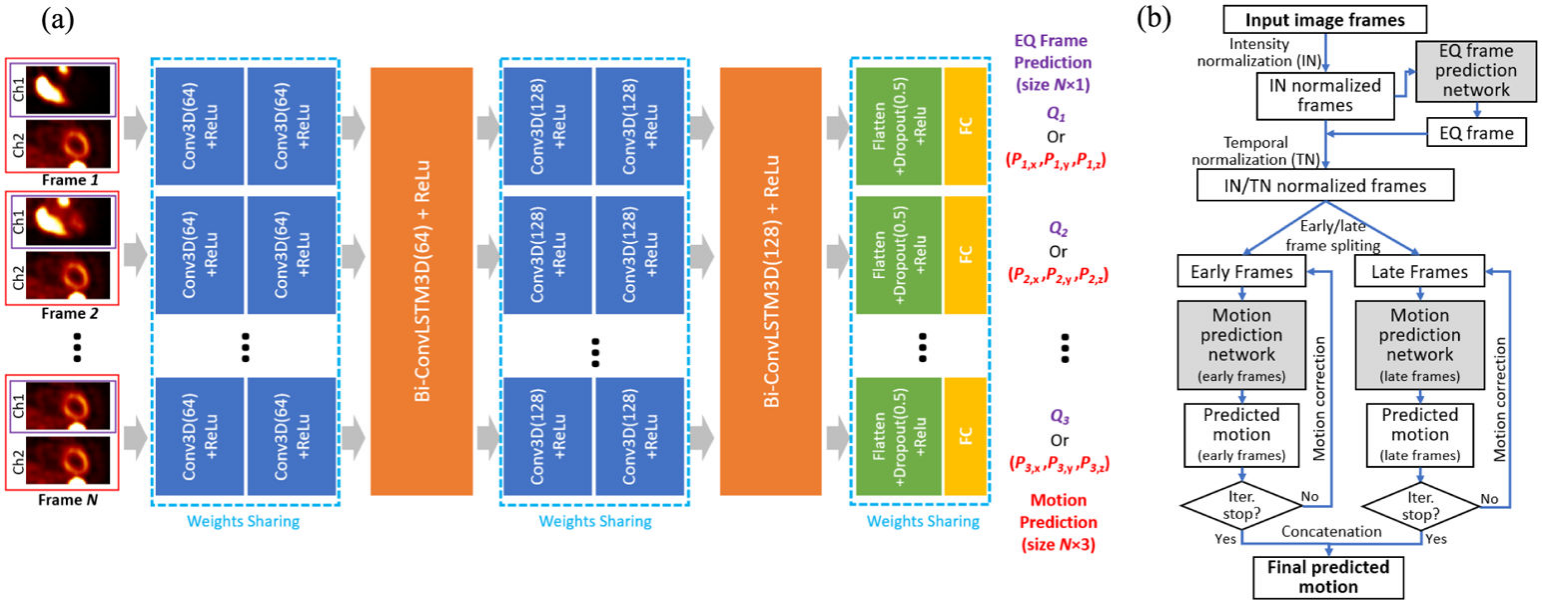
(a) The architecture of the motion prediction and EQ frame prediction network. The input frame images are only displayed as 2D images, although they are actually 3D images, with dual-channels (red) for motion prediction network and single-channel (purple) for EQ frame prediction network. The motion prediction network and the EQ frame prediction network share the same network structure except for the last fully connected (FC) layer. They are trained separately. (b) A diagram of the entire motion prediction workflow.

Our architecture is depicted in Fig. 2 (a). Each 3D image frame is fed into two 3D convolutional layers (Conv3D) with 64 kernels for encoding the spatial feature. The Conv3D layers are all followed by a ReLU activation function (each blue box in Fig. 2 indicates a Conv3D + ReLu layer). The feature maps for all the image frames are then sent to a bidirectional ConvLSTM3D unit for processing. This Conv3D plus bidirectional ConvLSTM3D combination was then repeated, with 128 kernels in each layer. Lastly, the feature map for each frame is flattened, followed by a dropout layer with ratio 0.5 and ReLu, and finally passed to a fully connected (FC) layer to regress the predicted motion displacement vector ***P***_*n*_ = (*P_n,x_*, *P_n,y_*, *P_n,z_*), where *P_n,x_*, *P_n,y_* and *P_n,z_* are the predicted motion displacements in the left-right (x), anterior-posterior (y) and superior-inferior (z) directions for each frame, *n* (1 ≤ *n* ≤ *N*) is the frame index and *N* is the total number of frames.

We used a stride of 2 in all the Conv3D layers instead of using max pooling, because max pooling has the well-known property of being local shift invariant to the small changes in the input [35]. In our application we do not seek for this property since our goal is to design a network sensitive to small changes caused by motion.

### D. Image Pre-Processing

For each dynamic image sequence, a median filter with window 3 × 3 × 3 was applied to each frame for noise suppression. The median filter was only applied for motion detection purpose. The detected motion was later applied to images without the filtering, to be consistent with the clinical filter settings. In addition, the following steps were applied.

#### 1) Dual-Channel Input:

In order to ensure the reference frame’s information was not lost along the information flow of the LSTM network, the reference frame (the last frame) was concatenated to each frame’s image as a second channel of the input (see Fig. 2 (a)), to provide a consistent motion estimation reference.

#### 2) Image Intensity Normalization:

The tracer activity level in our regions of interest (blood pools and myocardium) can vary a lot between frames, especially during the early frames. This can cause unstable training of the neural network. Therefore, each frame (cropped to 64 × 64 × 36) was normalized by the mean activity of the cropped region. Here, mean normalization was chosen over maximum normalization for being less sensitive to noise [25].

#### 3) Temporal Normalization:

**Fig. 3.**
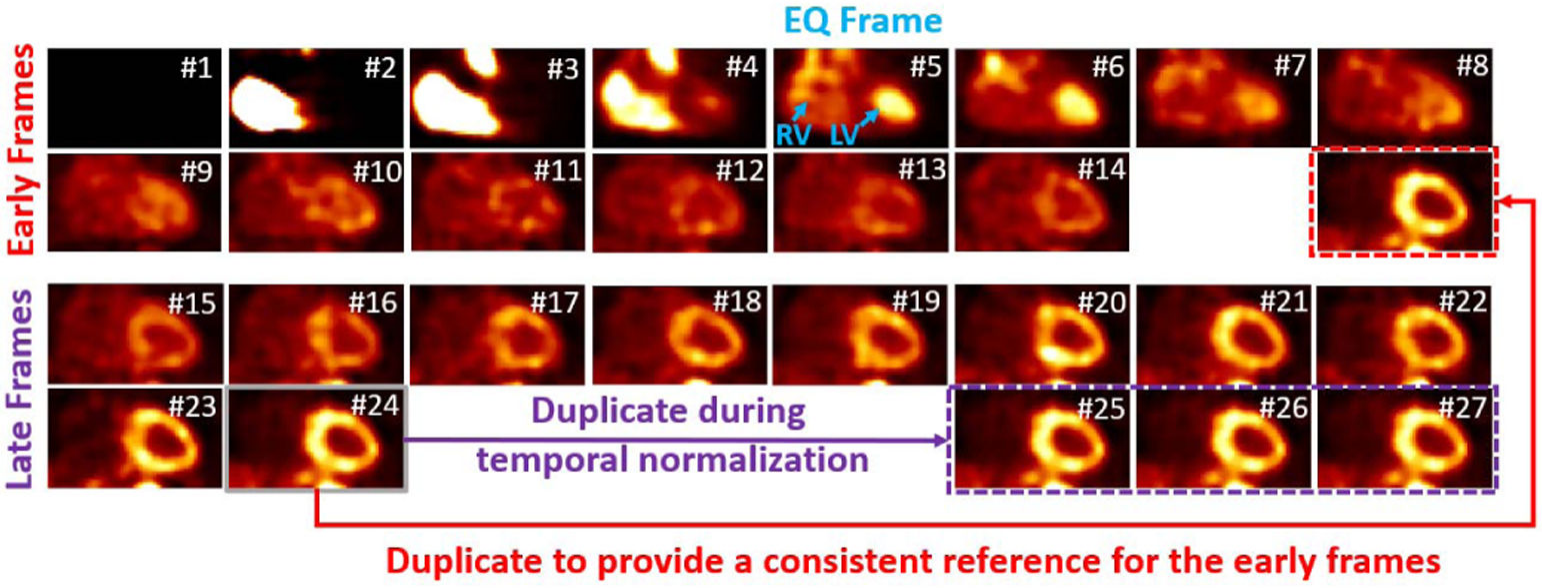
An example of the early frames and late frames after intensity and temporal normalization. The whole sequence was shifted towards the early phase by three frames, therefore the first three frames with zero activity were discarded, and the last frame was duplicated three times at the end of the late frames (purple). The last frame was also put at the end of the early frames to provide a reference (red). All the images are shown in the coronal view. The frame number after temporal normalization was shown for each frame. The EQ frame was pointed out in blue color.

After tracer injection and the start of the scanning, the time for tracer to reach the left ventricle blood pool can be different for each patient, which is another factor that could unstabilize the neural network’s performance. We defined equal (EQ) frame (with frame index *n*_*EQ*_), which is the first frame in which the activity in LV blood pools is equal or higher than that in the RV blood pool (see Fig. 3), to be used for temporal normalization. For each study, we shifted the whole image sequences back and forth so that the EQ frame has the same frame index *n_REF_* in the shifted sequence. If *n*_*EQ*_ > *n*_*REF*_, the 4D image sequence is moved towards the early phase. To maintain the same number of total frames, a number of (*n*_*EQ*_ – *n*_*REF*_) frames with no or little activity at the beginning were discarded, and the same number of the last frame was duplicated and added to the end. If *n*_*EQ*_ < *n*_*REF*_, the image sequence is moved towards the late phase, with (*n*_*REF*_ – *n*_*EQ*_) all-zero frames added at the beginning and the same number of the frames discarded at the end. However, the latter case will result in losing the information of the frames at the end of sequence. We noticed that for all the studies we acquired, (*n*_*REF*_ – *n*_*EQ*_) never exceeds two when *n*_*REF*_ = 7 was used and the first two frames always have zero or minimal activity. Therefore, we discarded the first two frames and duplicated the last frame twice for all the image sequences beforehand, to make sure no useful information was lost during the temporal normalization. We also set any frame before the EQ frame, with a total activity less than 1/10 of the EQ frame, to zero since these frames provide no useful information for motion estimation.

To automatically identify the EQ frame, the same network architecture in section II.C was used, except for the output layer. For the input of *N* frames, the output is a probability vector ***Q*** = (*Q*_1_, *Q*_2_, … *Q_N_*), as shown in Fig. 2 (a), where $Q_{n}\in R^{+}$ is the probability that the *n*-th frame is the EQ frame. The index *n*_*EQ*_ = argmax_1≤*n*≤*N*_ *Q*_*n*_ indicates the EQ frame. All 27 frames were used to predict the EQ frame, and only single channel images were used as the inputs (as shown by CH1 in Fig. 2 (a)).

#### 4) Early and Late Frames:

The tracer distributions are dramatically different between the early and late frames. Therefore, to let the network focus on the unique time dependent problems and to yield better performance, two networks with the same architecture (as described in section II.C) were trained independently for the early and late frames. After temporal normalization, all the frames were approximately cut into halves: the first 14 frames are considered the early frames and reflect the input function and initial myocardial extraction, and the last 13 frames are considered late frames and primarily reflect myocardial uptake and retention. The reference frame (the last frame) was also added to the end of the early frames set to provide a consistent reference, so that the early frames include 15 frames in total. An example of the early and late frames is shown in Fig. 3. The images are pre-processed with the image intensity normalization and temporal normalization.

During motion prediction, two network’s results were combined to provide the motion estimation for the whole image sequence.

### E. Network Training

To train the motion detection network for either early frames (15 frames in total) or late frames (13 frames in total), a total of 1 million motion replicate samples were randomly simulated based on the 65 patient scans described in section II.A. The training process was stopped after running through the 1 million examples. For each sample, one type of motion (square, triangle or spike) was randomly selected with a chance of 1/3. The number of motion frames were randomly selected between 2 and 7 with equal chances. Motion was randomly added anywhere except for the first 3 frames with negligible tracer activity (after temporal normalization) and the last reference frame. The maximum motion shift was limited to (4, 4, 4) voxels. For each axis, the direction and motion magnitude were randomly chosen with equal chances. For square motion, one set of motion shift and direction was simulated and used for all the selected frames; for triangle motion, one set of motion shift and direction was simulated and used as the maximum motion shift, with the other frames’ magnitude calculated with basic properties of triangles; for spike motion, the motion magnitude and direction for each selected frame was simulated independently. To further augment the data, an initial window shift was applied to all of the frames in each sample before the motion was added, and the maximum shift was limited to 3 voxels along each axis. For each sample, the whole sequence was also randomly moved towards the early phase or late phase up to 1 frame to provide data augmentation in the temporal dimension. All the randomness described above follows uniform distribution. The batch size for training was 32. The mean square error (MSE) loss was used to update the network. The Adam optimizer with an initial learning rate of 0.001 was used, where the learning rate decayed by a factor of 0.999 for every 10 batches trained.

Training the EQ frame prediction network is similar to training the motion prediction network, except that all the original 27 frames were used as the inputs and the cross-entropy loss was used instead of MSE loss. The temporal data augmentation can be up to 3 frames towards either the early or the late phase. Moreover, only 100,000 samples were generated and the learning rate decay factor was changed to 0.998, because this is a much less challenging problem compared to motion prediction hence fewer samples are needed. The frameworks were implemented using PyTorch and trained on a NVIDIA Quadro RTX 8000 GPU.

### F. Iterative Motion Correction

Once the motion estimation network was trained, motion correction was achieved by reversely shifting the cropping window in the original image space, based on the predicted motion. An iterative motion correction (IMC) strategy was applied during evaluation and testing, to ensure any residual motion can be detected and corrected: the corrected sequence from the previous iteration was fed into the network again for motion detection and correction in the next iteration. The iterative process stops until the iteration number exceeds 5 or the sum of the detected motion magnitudes of all the frames for the current iteration is smaller than 0.1 voxels, whichever comes first. The final motion estimation is the concatenation of the estimated motion for the early and late frames. The entire motion prediction workflow is shown in Fig. 2 (b).

### G. Comparison With Conventional Registration Approaches

The proposed DeepMC was compared with conventional registration methods. Two registration strategies were evaluated, namely individual registration (IR) and chain registration (CR). Individual registration is the most straight forward approach: each individual frame was directly registered to the reference frame (the last frame). However, this might not be a fair comparison since the early frames are substantially different from the reference frame, which could cause unsatisfactory or even failed registration. Therefore, we also implemented chain registration, where starting from the second to last frame, each frame was registered to its next frame. Since registration was only performed between two adjacent frames that share a lot of similarities, superior performance might be expected in the presence of tracer distribution change between frames.

The SimpleElastic library was used for both registration approaches. Only rigid translational motion was considered, and the ‘Advanced Mattes Mutual Information’ metric was used. The ‘Number of Resolutions’ was set to 3. Other parameters were used as default and can be found at [36]. Same as DeepMC, a median filter was applied to each frame in advance for noise suppression.

### H. Comparison With Other Neural Networks

The proposed DeepMC was also compared with another convolutional neural network (CNN). We chose a recent CNN architecture [37] that was designed for estimating rigid motion between two 3D image volumes. We made a few slight changes to the network structure, and the details about this network can be found in the supplementary material. This network can be viewed as a CNN-based image registration method, although without the LSTM component.

Similar to the conventional registration methods, we applied the CNN-based method through two strategies. In the first strategy, the CNN was trained and applied between each frame and the reference frame for motion estimation. In the second strategy, motion estimation was performed in a chain fashion, where the CNN was trained and applied between each frame and its next frame. We refer to these two methods as MC-CNN and MC-CNN-C (C stands for chain) from now on. For fair comparisons with DeepMC, we adopted the same convention to divide the image sequences into early and late frames for both MC-CNN and MC-CNN-C. Image normalization was also applied beforehand, although iterative motion correction (IMC) was not used because we found IMC resulted in slightly worse results here. More training details can be referred to the supplementary material.

### I. Evaluation

To evaluate the performance of DeepMC and the other approaches, for each one of the 20 scans described in Table I, 10 motion replicates were simulated for each type of the square, triangle and spike motion types, resulting in a total of 600 motion affected samples. The motion simulation process was similar to the process described in section II.E, except that: 1) a random number between 5 and 11 of frames were added with motion; 2) for each sample, motion was added to the entire sequence (except for the first 3 frames and the last reference frame), instead of adding motion to early frames and late frames separately; 3) the initial random window shift was not applied.

The motion estimation was evaluated in terms of motion estimation mean error and maximum error across all the frames. The mean error was calculated as $\frac{1}{N-3}\sum_{n=3}^{N-1}\sqrt{(P_{n,x}-M_{n,x}{)}^{2}+(P_{n,y}-M_{n,y}{)}^{2}+(P_{n,z}-M_{n,z}{)}^{2}}$ where (*P_n,x_*, *P_n,y_*, *P_n,z_*) and (*M_n,x_*, *M_n,y_*, *M_n,z_*) are the motion prediction and ground-truth motion vectors in three directions (*x*, *y*, *z*) for frame *n*, and *N* = 27 is the total number of frames. The first 2 frames were not included in the evaluation for having no or little activity and the last (reference) frame was also not included. The maximum error was calculated as $max_{n=3}^{N-1}\sqrt{(P_{n,x}-M_{n,x}{)}^{2}+(P_{n,y}-M_{n,y}{)}^{2}+(P_{n,z}-M_{n,z}{)}^{2}}$, which reflects the performance limitation of the motion estimation.

Additionally, both the LV blood pool image-derived input function (IDIF) and LV myocardium TACs were fit to a 1-tissue (1T) compartment model to obtain estimates for uptake rate *K*_1_ corrected with LV blood volume and spillover term [38]. Weighted least squares (WLS) fitting was used to estimate the parameters. The weights were calculated as [38]: $w_{n}=L_{n}^{2}∕(T_{n}\times DCF^{2})$, where *L*_*n*_ is the *n*^*th*^ frame duration, *T*_*n*_ is the total activity for the *n*^*th*^ frame and *DCF* is the decay correction factor. The IDIF was estimated from a rectangular VOI manually placed towards the base of the LV (average size 3.9 cm^3^ for all the 30 scans in the evaluation, including 20 motion-free scans and 10 scans with real motion) for each scan. The entire LV myocardium was manually segmented (average size 130.3 cm^3^ for all the 30 scans) to measure the LV myocardium TAC. Myocardial blood flow (MBF, in the unit of mL/min/g) was then computed from the estimated *K*_1_using a previously validated relationship (using scale-uncorrected IDIF) [39]. For the motion-corrected (using the proposed method and the two registration approaches) and uncorrected images, the MBF percentage biases were calculated using the MBF values obtained on motion-free images as the ground-truth: (*MBF*_*M*_ – *MBF_MF_*)/*MBF*_*MF*_×100%, where *MBF*_*M*_ represents the MBF estimated on the motion uncorrected or corrected images, and *MBF*_*MF*_ were estimated on the motion-free images. In addition, the weighted sum-of-squared (WSS) residuals (the residuals between the MBF model solutions and the measured TACs) from the WLS fittings were also calculated for each group. Since the weights *w*_*n*_ have a unit of min^2^/Bq × mL, the WSS residuals have a unit of (Bq/mL)^2^ × min^2^/Bq × mL = Bq/mL×min^2^.

For the 10 testing patient scans with real motion, although they were selected based on the manually estimated motions by the clinical staff using the 4DM software, these estimates were not exportable and were based on the resliced image views, whereas in our framework motion was estimated in the original transaxial image views. Therefore, due to the lack of the motion ground-truth in our study, the percent difference between MBFs measured on the images before and after motion correction were reported. The percent difference was calculated as 2(*MBF*_*UC*_ – *MBF*_*MC*_)/(*MBF*_*UC*_ + *MBF*_*MC*_)×100%, where *MBF*_*UC*_ and *MBF*_*MC*_ represent the MBF estimated on the motion uncorrected and motion corrected images, respectively. MBF for the region supplied by the right coronary artery (RCA) (average size 58.3 cm^3^) was also calculated in addition to the global MBF calculated on the whole LV myocardium. In this study, we focused on RCA in the regional MBF investigation as studies have shown that RCA is substantially more susceptible than left anterior descending (LAD) and left circumflex (LCX) arteries to motion induced MBF errors [40]. The WLS fitting residuals derived from the voxels within the whole LV myocardium and RCA were also calculated for these 10 patient scans.

## III. Results

### A. EQ Frame Prediction

The EQ frames *n*_*EQ*_ (defined in section II.D.3) range between 5-11 for the 20 evaluation scans and 5-10 for the 10 testing scans, based on our manual labeling. The EQ frame prediction network trained over the 65 training scans correctly identified the EQ frames for all of these 30 scans, an accuracy of 100%.

### B. Motion Characteristics in the Evaluation With simulated Motion

**Fig. 4.**
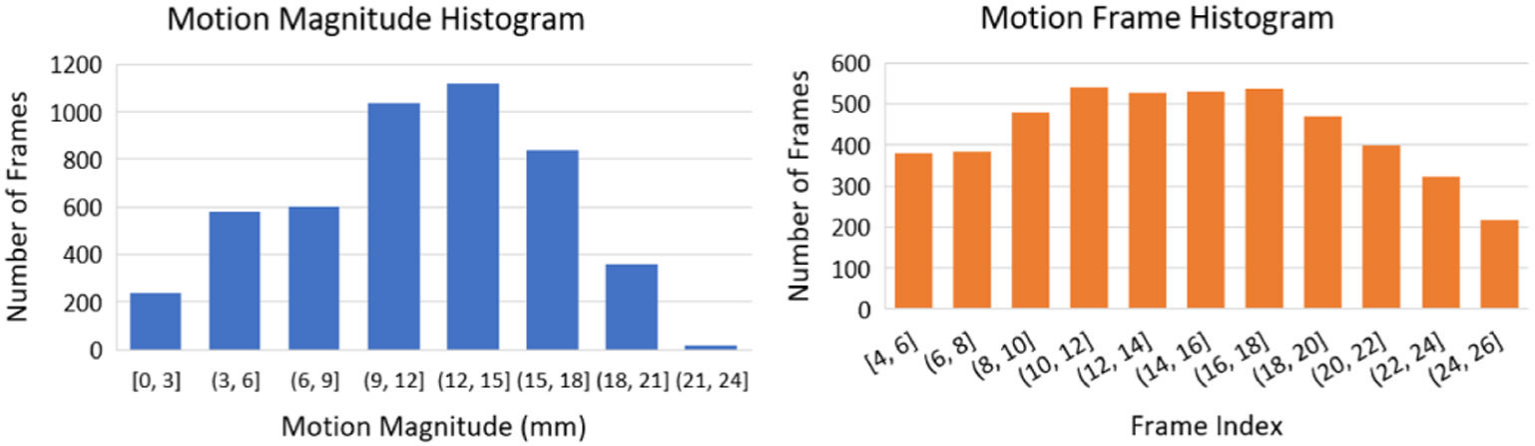
The distribution of the motion magnitude (mm) and the frame index of the motion-affected frames for the 600 image sequences in the evaluation.

For the 600 image sequence samples used in evaluation with simulated motion, a total of 4806 frames were added with simulated motion based on the criteria described in section II.I. Fig. 4 shows the distribution of the motion magnitude (mm) and the frame index of these motion-affected frames. From the motion magnitude histogram, it can be observed that the majority of the simulated motions range between 9-18 mm, which is similar to what was observed in [4] where a significant fraction (38%) of the observed motions were severe motion (7-18 mm) in a study with 236 patients. The frequency of motions also roughly has a uniform distribution across all the frames, as can be seen in the motion frame histogram.

### C. Ablation Study

An ablation study was conducted to demonstrate the effects of different image pre-processing components in the proposed framework. The ablation study results are given in Table II. It can be seen that, when all the image pre-processing methods described in section II.D were applied, the lowest motion prediction errors were achieved in terms of both mean motion error and max motion error.

Particularly, the temporal normalization (TN) resulted in the largest improvement. Two groups of studies were compared: TN was applied in neither training nor evaluation data, and TN was not applied in training data but applied in evaluation data. As can be seen, when TN was applied during neither training nor evaluation, the worst results were obtained. Although applying TN on the evaluation data further helped, applying TN on both training and evaluation is recommended.

Using the reference frame as the second channel for each frame also substantially improved the results. Note that the results from using only single channel are still reasonable, though not optimal, suggesting that the LSTM architecture was able to pass the information of the reference frame nonlocally throughout the sequence. The iterative motion correction (IMC) brought a moderate improvement, indicating that the majority of the motions were successfully detected in the first pass. On average, the iterative process stopped at iteration number 2.61 ± 1.02 for the early frames and 1.90 ± 0.75 for the late frames, suggesting that the early frame motion is slightly more challenging to be detected compared to the late frame motion. The intensity normalization also moderately improved the motion detection performance.

Using a validation dataset can potentially help reduce overfitting and identify the best hyperparameter settings (in our case, the hyperparameter we want to optimize is whether or not we should apply each image pre-processing step as described in Section II.D). Therefore, we further randomly divided the original training scan set (from 65) to 55 for training and 10 for validation, and used the validation error to identify the best model for each group in the ablation study. The best models identified by the validation set were evaluated on the same evaluation set with 600 motion replicates. The validation studies confirmed that the strategies presented in this paper are indeed optimal by incorporating all these image-preprocessing steps. However, we also found that using the validation set resulted in larger motion estimation errors for most of the groups, likely because of the reduction of the training scan set. This suggests that using a validation set can be helpful when there is an abundant training set, but might cause the opposite effect with a limited training set. We only evaluated the model trained without the validation set in the remaining of this paper. For more details about training and results please refer to the supplementary material.

### D. Evaluation of Motion Estimation Performance

**Fig. 5.**
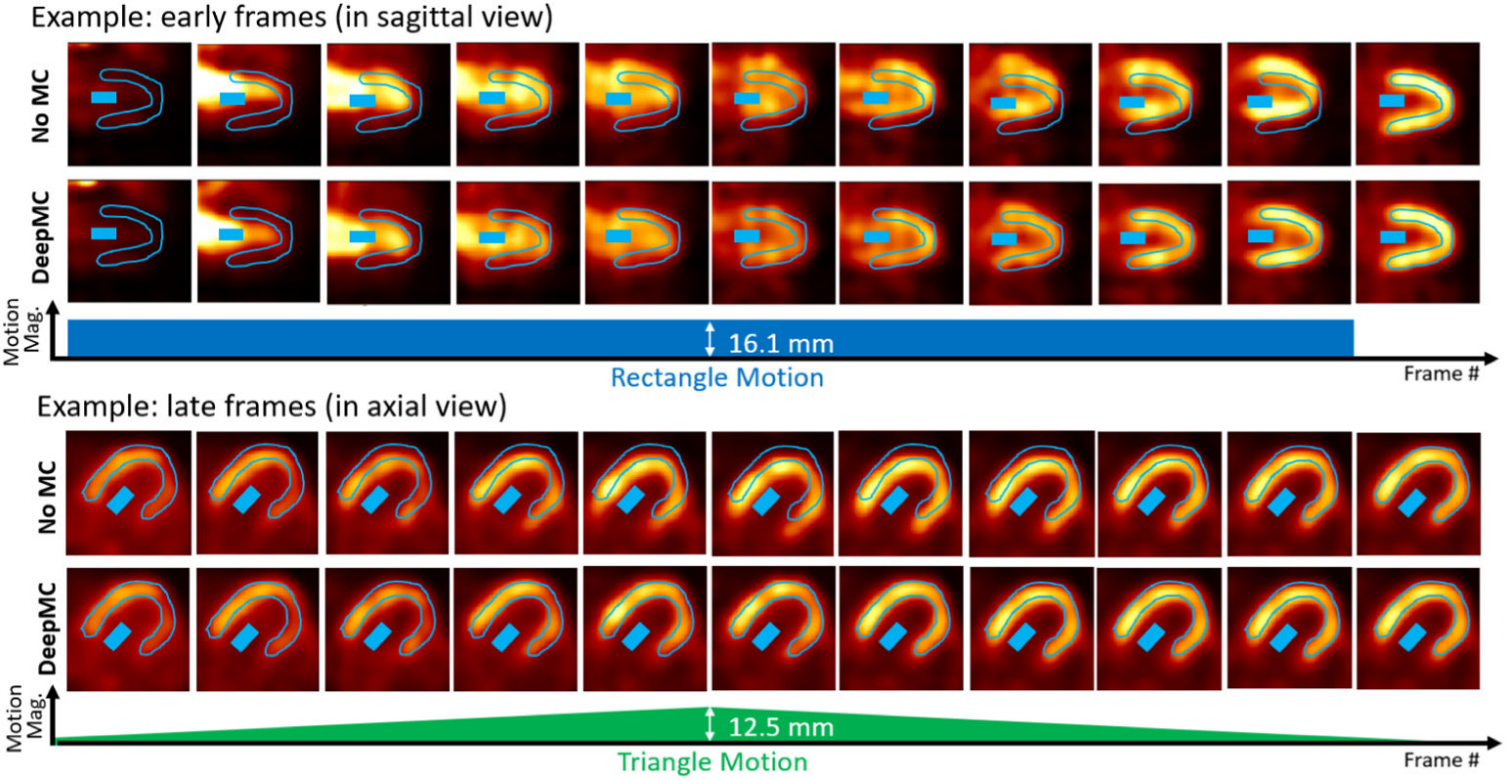
Two examples (selected from the 600 evaluation cases) of the image frames with simulated motion (No MC) and after motion correction (DeepMC). The two examples correspond to two different patients. The images were displayed after intensity normalization. The top example shows the early frames with rectangle motion (motion magnitude 16.1mm), and the bottom example shows the late frames with triangle motion (peak motion magnitude 12.5mm). In both examples, the LV myocardium and LV blood pool ROIs drawn on the last frame are displayed on each image frame.

Fig. 5 shows two examples (selected from the 600 evaluation cases) of the image frames with simulated motion (No MC) and after motion correction (DeepMC). The first example shows the early frames with rectangle motion (motion magnitude 16.1 mm) in sagittal view, and the second example shows the late frames with triangle motion (peak motion magnitude 12.5 mm) in axial view. In both examples, the LV myocardium and LV blood pool ROIs drawn on the last frame are displayed on each image frame. It can be seen that without motion correction, motion caused mis-alignments between the ROIs and the actual LV myocardium and blood pool. In comparison, DeepMC can provide excellent motion correction consistently across all the image frames.

**Fig. 6.**
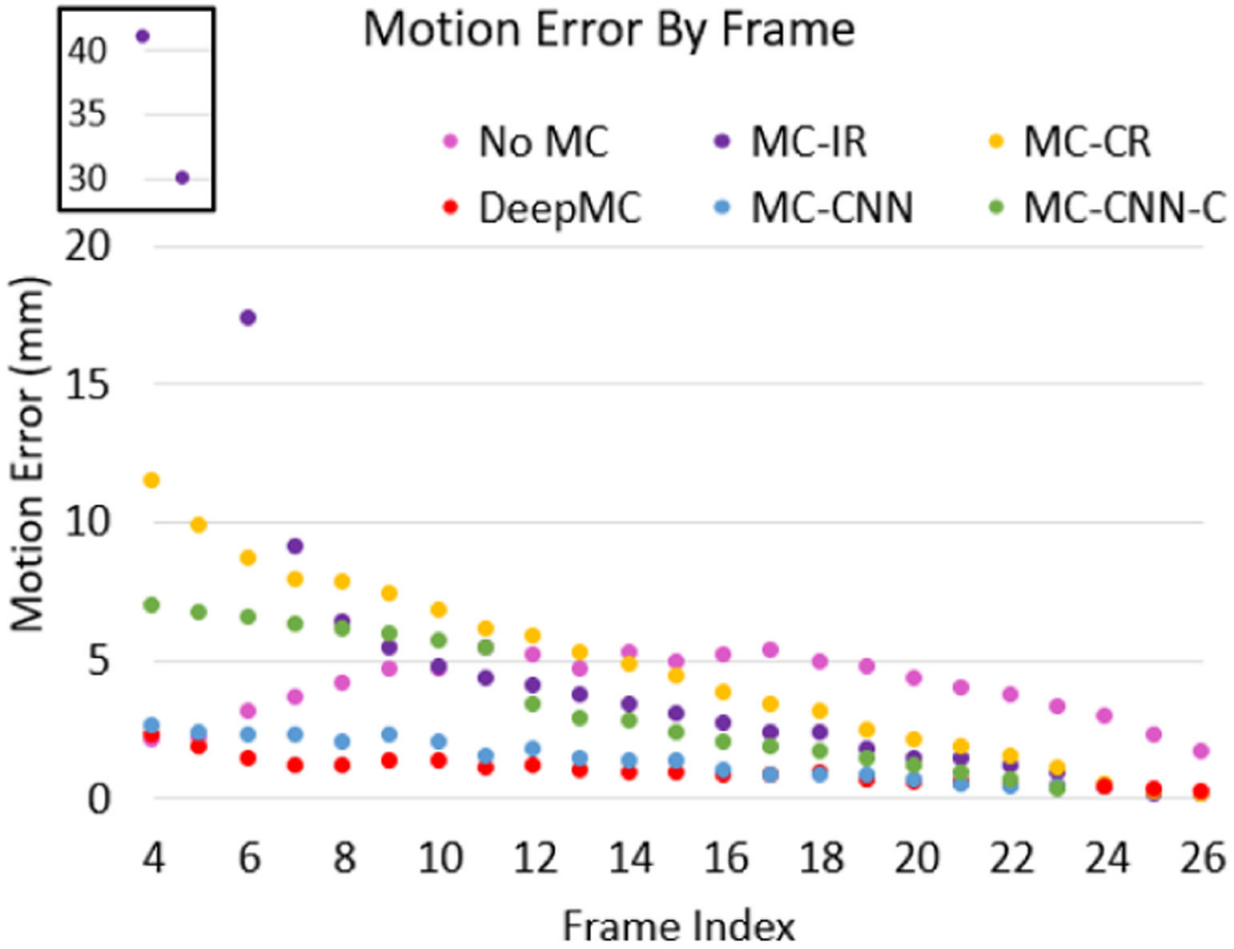
The average motion estimation error (mm) by frame for the different methods on the 600 evaluation image sequences. The first two data points of MC-IR with large motion errors are shown in a zoomed region for better illustration.

Fig. 6 shows the average of the motion estimation error (mm) as a function of frame index for the motion correction with DeepMC, individual registration (MC-IR), chain registration (MC-CR), CNN (MC-CNN), chain CNN (MC-CNN-C) and no motion correction results on the 600 evaluation image sequences with simulated ground truth motion. As can be seen, although the individual registration performed well for the late frames, it completely failed for the early frames, due to the substantial tracer distribution difference between the early frames and the late reference frame. The chain registration only performed better than the individual registration for the early frames, but the errors for the early frames are still much larger than those without applying motion correction. A clear trend of increasing error from the late phase to the early phase can also be observed, suggesting that the registration error can accumulate for the chain registration. The chain CNN also presented a trend of error increase towards the early frames, and the results are slightly better than the conventional registration methods. In comparison, the CNN performed much better than the chain CNN. The proposed DeepMC obtained the lowest error level overall, especially in the challenging early blood pool phase and the transition phase.

The proposed DeepMC was also much faster computationally as compared with both conventional registration methods. The computation runtime on the 600 cases (23 registrations per image sequence) for each registration method was more than 20 hours, whereas the proposed DeepMC approach only took less than 40 min using a batch size of 1. The CNN methods were slightly faster than DeepMC, which took about 30 min.

A comprehensive summary of the evaluation results is shown in Table III. In terms of all the error types, the individual registration method (MC-IR) resulted in the worse results among all the methods. The chain registration (MC-CR) was superior to MC-IR, but was overall no better or even worse than the results without applying motion correction, mainly due to the large motion estimation errors in the early phases (see Fig. 6). Both the CNN (MC-CNN) and the chain CNN (MC-CNN-C) outperformed the conventional registration methods. Particularly, the CNN performed much better than the chain CNN in terms of motion estimation error and MBF bias, but interestingly produced larger fitting errors than the chain CNN. Note that this is consistent with the conventional registration, where the fitting residuals from the chain registration was also smaller than the individual registration. The proposed DeepMC method obtained the lowest motion estimation errors and MBF biases for all the three motion types. The weighted fitting residuals for the DeepMC method were also the lowest among all the comparison groups and are highly consistent with the fitting residuals of the motion-free images.

**Fig. 7.**
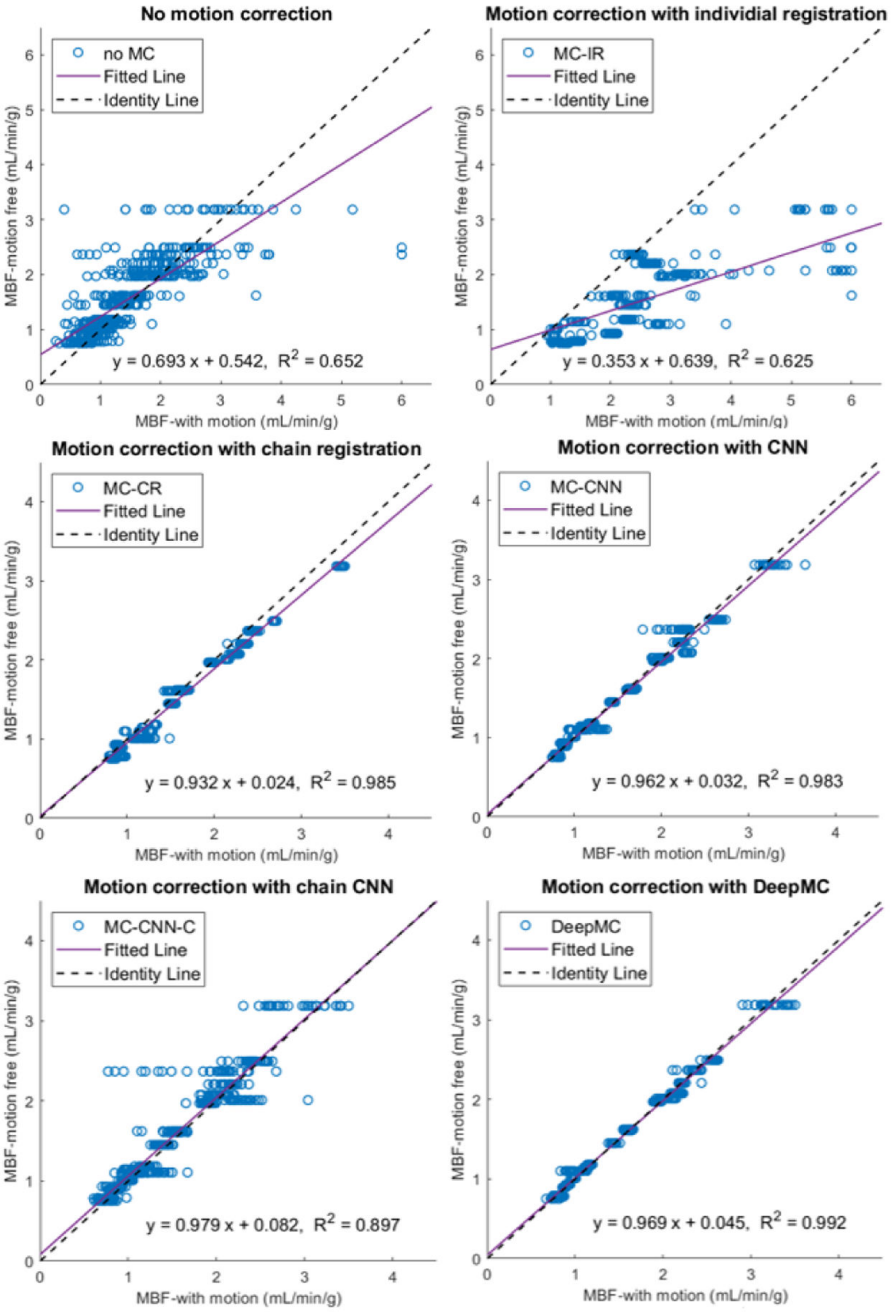
The scatter plots of the MBF estimation between the motion-corrected and motion-free groups. Each one of the parallel clusters (20 in total) represents the results from the 30 motion replicates derived from each of the 20 motion-free patient scans.

**Fig. 8.**
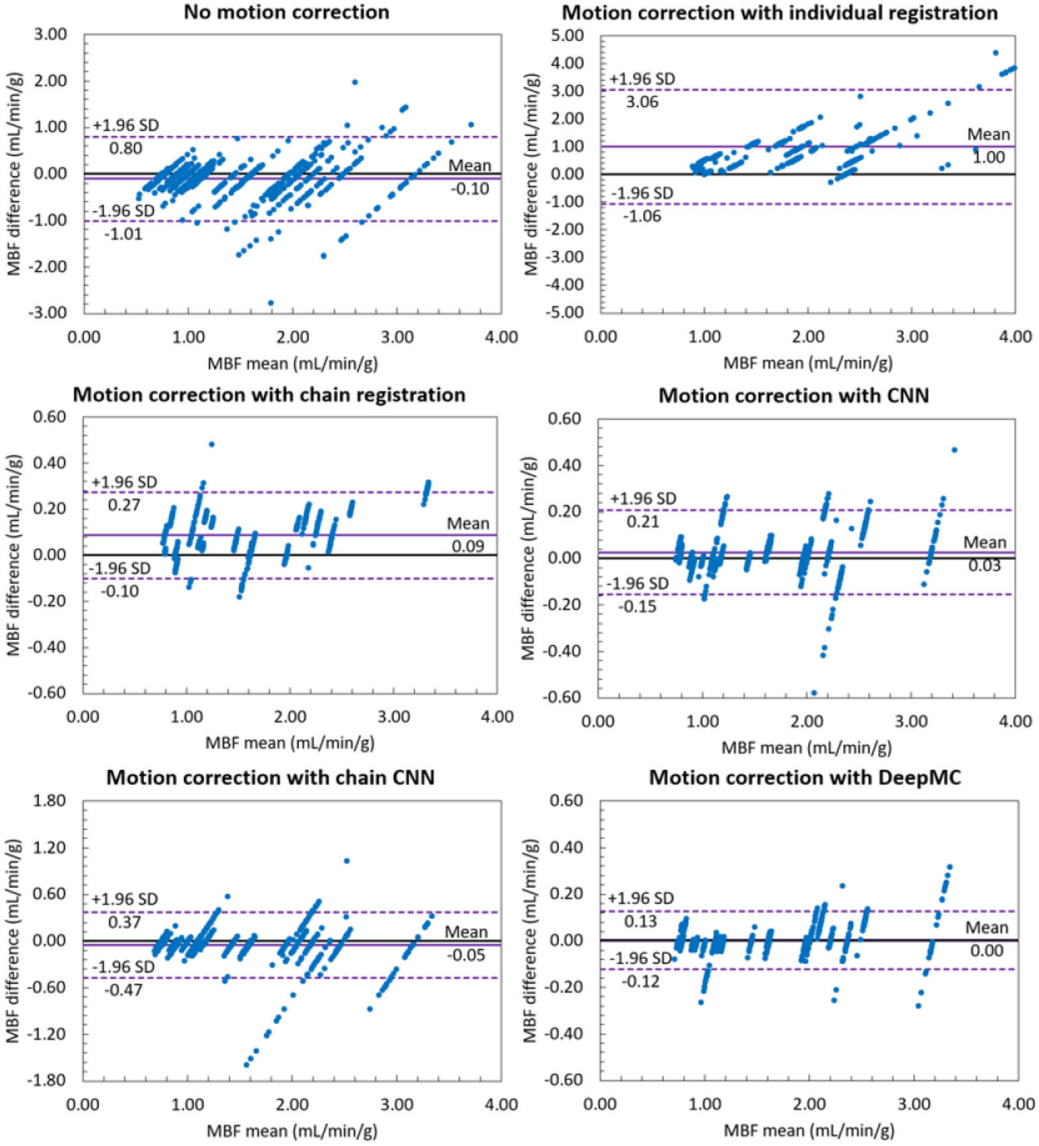
The Bland-Altman plots of the MBF estimation between the motion-corrected and motion-free groups. Each one of the parallel clusters (20 in total) represents the results from the 30 motion replicates derived from each of the 20 motion-free patient scans. Note the scale difference of the y-axis for different groups.

The MBF bias for each evaluation sample can be positive or negative, therefore the mean values of the MBF bias across all the samples indicate if there is a systematic bias, whereas the standard deviation shows the averaged absolute MBF bias. From Table III it can be observed that both conventional registration-based methods and both CNN-based methods resulted in systematic biases in MBF measurement. After motion correction using DeepMC, both the mean and standard deviation of the MBF bias caused by motion were reduced to a minimal level. Fig. 7 and Fig. 8 show the scatter plots and Bland-Altman plots of the MBF estimation between the motion-corrected and motion-free groups. Similar to our previous observations, the proposed DeepMC obtained the best performance among all the compared methods and obtained highly consistent MBF results with the motion-free results.

### E. Patient Evaluation With Real Motion

**Fig. 9.**
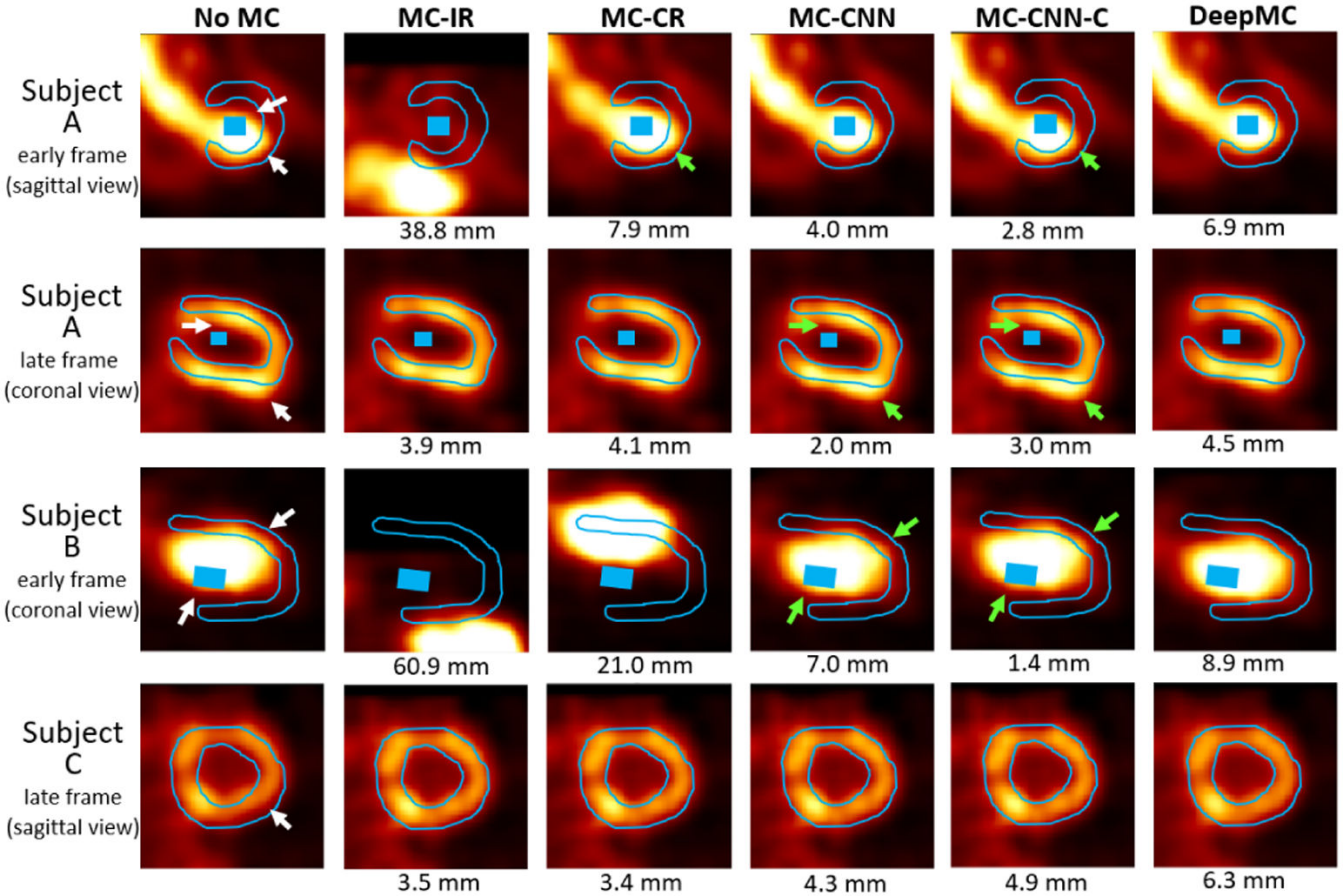
Examples of the image frames from three subjects before and after motion correction selected from three patient scans. The LV myocardium and blood pool ROIs drawn on the last frame were overlaid with the images (the blood pool ROI was not shown on the selected slice for Subject C). The white arrows pointed out the mismatches caused by motion when no motion correction was applied (noMC). The green arrows pointed out the residual mismatches after motion correction in some of the results. The motion magnitudes detected by different motion correction methods were also shown beneath the images.

As was explained in section II.I, due to a lack of the motion ground-truth, the motion correction for the patient studies with real motion was evaluated qualitatively and semi-quantitatively. After motion correction using our proposed method, most of the motions were correctly compensated by visual checking of the dynamic series, although underestimated motion was found for a few frames due to the low image quality caused by intra-frame motion or image noise. Examples of the early and late frames before and after motion correction selected from three patient scans were given in Fig. 9. The LV myocardium and blood pool ROIs drawn on the last frame were overlaid with the images. The white arrows pointed out the mismatches caused by motion, which were successfully corrected by the proposed DeepMC method. Residual mismatches can be observed in the images from all the other methods (pointed by the green arrows), which were caused by either over-estimated or under-estimated motion, as can be seen from the detected motion magnitude beneath each image (using DeepMC as reference).

Table IV summarizes the MBFs and fitting residuals measured on the whole LV myocardium and the RCA ROIs for different motion correction methods. Both DeepMC and MC-CNN obtained smaller fitting residuals than the fitting residuals without motion correction (No MC), although the fitting residuals from MC-CNN was smaller than those from DeepMC. Nonetheless, we recommend only using the fitting residuals as a reference for evaluation, as the fitting residuals alone does not necessarily imply motion correction quality. For example, in Table III, MC-CNN obtained smaller motion estimation error and MBF bias compared with MC-CNN-C, albeit larger fitting residuals. Without the motion ground-truth, visual checking might be more suitable. The visual observations (as shown in Fig. 9) suggest that DeepMC obtained better motion correction results than the other methods, and we only focus on DeepMC’s results from now on.

**Fig. 10.**
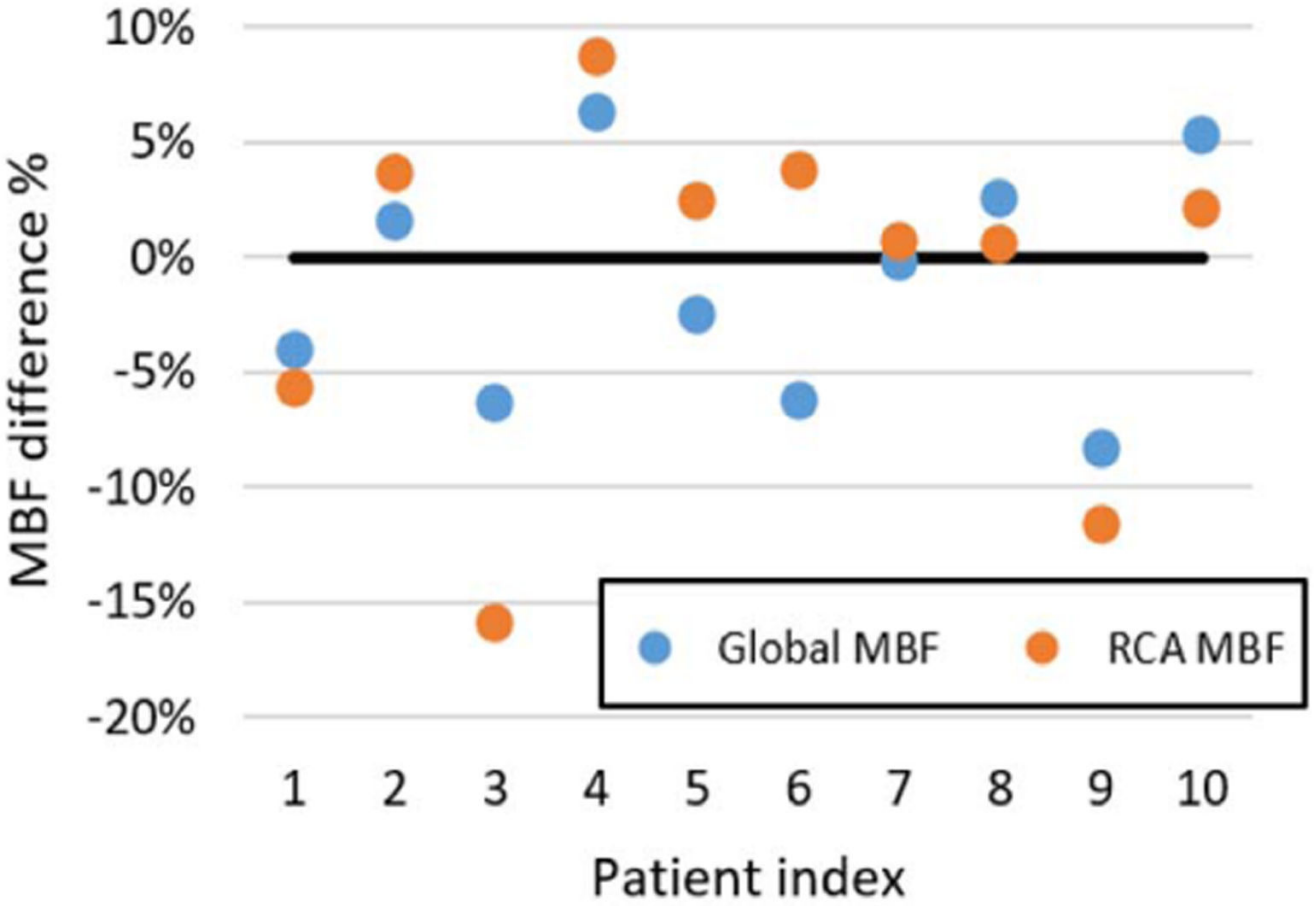
Scatter plots of the percentage difference between the MBFs measured on the images before and after motion correction using DeepMC.

For DeepMC, on average 7.9 ± 2.1 frames with motion larger than 3 mm were identified for each subject, and the averaged detected motion within those frames with motion larger than 3 mm was 4.9 ± 0.6 mm. Fig. 10 shows the percentage difference between the MBFs measured on the images before and after motion correction using DeepMC. From the scatter plot, it can be seen that the RCA MBF differences have a wider distribution compared with the global MBF differences. An average of 4.4% ± 2.4% and 5.5% ± 4.8% absolute percentage difference before and after motion correction were measured for global MBF and RCA MBF, respectively. This suggests that the RCA MBF is more susceptible to the effect of motion. Note that although median or severe motion were identified in all of these cases, the global MBF and RCA MBF differences before and after motion correction were not always large.

Particularly, in the examples shown in Fig. 9, the first two rows are images from the same Subject A, where the detected motions by DeepMC in the shown early and late frames were 6.9 mm and 4.5 mm, respectively. The global MBF and RCA MBF differences before and after motion correction were 8.3% and 11.7%, respectively. The shown early frame in Subject B corresponds to a detected motion of 8.9 mm, and the late frame in patient Subject C corresponds to a detected motion of 6.3 mm. However, the global MBF and RCA MBF differences before and after motion correction were 6.3% and −3.7% for Subject B, and −1.6% and −3.7% for Subject C. As can be seen, not all motions have a large impact on MBF estimation.

## IV. Discussion

Compared with other medical image registration problems where the target image and reference image share a lot of similarities, motion detection and correction in dynamic images is much more challenging since the target image can be dramatically different from the reference image due to the rapid tracer kinetics. Therefore, we proposed to use a 3D bidirectional-LSTM neural network to better utilize both local and nonlocal temporal information in the 4D dynamic image data for motion detection. The proposed network is capable of learning the typical spatial trace distribution change in time (also indicated by the ability to detect the EQ frame), which can help motion estimation. Multiple image-preprocessing methods customized to our unique problem were also designed to further boost the motion detection performance. Due to the lack of the ground-truth motion, we trained and evaluated the network based on selected motion-free patient data incorporated with various types of simulated motion. Compared with the conventional registration methods, the proposed DeepMC method obtained substantially lower motion estimation error and more consistent MBF estimations with the motion-free results. In addition, the qualitative evaluation on 10 patient datasets with clinical observed real motion also demonstrated the effectiveness of the proposed method.

Training the network took about 40 hours each for the early phase and late phase. However, once the networks were trained, processing one dynamic image sequence only took about 4 seconds, which is 30 times faster than the registration-based methods. Since the proposed method was a post-processing method that applies on the already-reconstructed images and does not rely on the sinogram raw data, it can be easily integrated into the clinical workflow.

This work has several limitations that lead to future investigations. First, only rigid translation motion was considered in this study. At Yale New Haven Hospital, only translation motion was typically corrected manually, with occasional rotational motion correction. Correcting for rotation motion or even non-rigid motion can potentially further improve MBF quantification accuracy. Even though rotational and nonrigid motions can be simulated during training, depending on the severity of the rotational and nonrigid motions, whether it is necessary to consider these more complicated corrections needs further investigation due to the limited image resolution and the presence of noise in ^82^Rb PET studies. Additionally, to what extent our proposed DL method could be pushed for these more complicated and challenging motion estimations before breaking down is also of our great interest and will be explored in future studies. Second, this work focused on the inter-frame motion caused by the voluntary body motion and breathing pattern change of respiratory motion. However, the intra-frame motion caused by respiratory motion, cardiac motion and body motion can result in blurred or distorted frames, which might compromise the inter-frame motion estimation. Using frames with short durations might lead to improved temporal resolution with reduced intra-frame motion, which could potentially lead to better performance for DeepMC. However, shorter frame durations will also amplify image noise, which could affect motion estimation performance. Therefore, the trade-off between temporal resolution and signal-to-noise ratio needs to be further investigated. Alternatively, combining the proposed approach with other approaches that focus on intra-frame motion estimation and correction could further improve quantitative accuracy of MBF, and will be explored in the future. Third, in this paper we focused more on the technical development and demonstrated the feasibility of deep-learning based motion correction for dynamic cardiac PET, where evaluation was performed using motion-free patient data with simulated motion. The motion-free patient cases were selected visually, which may not be truly motion-free. In addition, the simulated motion might not mirror real clinical motion (e.g., the motion pattern or magnitude). For the small cohort of patient data with real motion, due to the lack of the ground-truth motion, the motion estimation results were only evaluated qualitatively and only the differences brought by the proposed motion correction was shown. Future work will include acquiring more patient data with manually labelled motions, and comparing the MBF with motion correction to clinical outcome data. Fourth, currently the CT-based attenuation correction was performed by manual registration between the CT and the static PET reconstruction. Misalignments between CT and each PET dynamic frame were not accounted for because frame-by-frame attenuation map realignment was not available in our PET/CT system. This can cause attenuation artifacts [41] on the dynamic frames and potentially impact quantification. Furthermore, the networks are being trained with simulated motion applied to motion-free patient cases, which as a result are not affected by this activity-attenuation mismatch. The trained networks might not expect this mismatch appeared on real patient cases and suboptimal performance can be expected. Preferably motion should be estimated from preliminary reconstructions without attenuation correction, and then the estimated motion parameters should be applied to the activity and attenuation pair to obtain fully motion-corrected activity reconstructions. Training and evaluating networks based on PET activity images before attenuation correction will be explored in the future studies. Fifth, in the current approach the image windows were cropped around the mid-point of the septal wall, which were all manually identified. However, this step can be easily achieved by designing an image-processing method or training another neural network.

In addition to the future works discussed in the previous paragraph, some other future directions are also worth exploring, including segmenting out the heart region first to exclude the influence of extra-myocardial activity, training and evaluation based on images in the resliced views and applying the proposed approach to dynamic imaging of other organs (for example, brain imaging) and dynamic SPECT imaging.

## V. Conclusion

This work presents an automatic motion correction framework for dynamic cardiac PET using deep learning. The proposed LSTM-based network can detect and correct inter-frame rigid motion for both early frames with fast tracer distribution change and later frames with slower tracer kinetics. Evaluation with patient data demonstrated the effectiveness of motion correction and improved accuracy of MBF quantification.

## Supplementary Material

supp1-3082578

## Figures and Tables

**TABLE I:** A Summary of the Characteristics of the Included Scans

	# of scans (rest/stress)	# of patients (male/female)	Age	BMI
			(Mean±Std)	
Training	65 (44/21)	45 (21/24)	62.2±11.7	38.3±11.4
Eval. w/sim. motion	20 (11/9)	14 (4/10)	65.4±9.5	33.6±8.2
Eval. w/real motion	10 (4/6)	9 (6/3)	69.0±12.1	34.9±8.7

**TABLE II:** Ablation Study Results in Terms of Mean and Max Motion Estimation Errors Across All the 600 Evaluation Samples. MC: Motion Correction; IN: Intensity Normalization; TN: Temporal Normalization; IMC: Iterative Motion Correction

	Mean Motion Err. (mm)	Max Motion Err. (mm)
No MC	3.99±1.67	14.85±4.09
Single Ch. Input	1.16±0.72	3.73±1.78
No IN	0.96±0.48	3.70±1.82
No TN (Eval. w/o TN)	2.50±1.94	22.27±16.36
No TN (Eval. w/ TN)	1.03±0.52	3.85±1.62
No IMC	0.99±0.63	3.92±1.98
All applied	0.92±0.51	3.48±1.94

**TABLE III:** A Comprehensive Summary of the Evaluation Results. For MBF Bias, * Indicates the Results’ Mean Is Significantly Different From Zero Based on Paired t-Test (5% Significance Level). Bold Font Indicates the Best Performer in Each Group

Error Type	Method	Square Motion(200)	Triangle Motion(200)	Spike Motion (200)
Mean Motion Error (mm)	NoMC	4.6±1.7	2.6±1.0	4.7±1.3
	MC-IR	6.4±2.2	6.4±2.2	6.4±2.1
	MC-CR	4.6±1.0	4.7±0.9	4.5±0.9
	MC-CNN	1.8±0.8	1.6±0.7	1.7±0.7
	MC-CNN-C	4.4±3.0	3.0±1.5	4.7±2.6
	DeepMC	**1.0±0.6**	**0.9±0.5**	**0.8±0.4**
Max Motion Error (mm)	NoMC	13.3±3.7	12.8±3.7	18.4±1.8
	MC-IR	46.7±19.7	46.4±19.9	46.2±19.2
	MC-CR	11.7±2.6	11.8±2.5	11.7±2.4
	MC-CNN	5.5±2.5	5.3±2.5	5.9±2.4
	MC-CNN-C	10.1±5.9	8.2±3.7	10.7±5.6
	DeepMC	**3.6±2.0**	**3.2±1.9**	**3.6±1.9**
MBF Bias (%)	NoMC	−9.8±33.0%*	−7.4±16.2%*	−4.0±17.3%*
	MC-IR	62.3±51.3%*	62.1±52.0%*	64.8±53.1%*
	MC-CR	6.0±7.4%*	5.9±7.3%*	6.1±6.9%*
	MC-CNN	1.2±5.9*	1.7±5.7*	2.2±5.6*
	MC-CNN-C	−2.3±10.4*	−2.8±12.5*	−3.0±10.6*
	DeepMC	**−0.5±4.6%**	**−0.1±3.9%**	**0.4±3.6%**
Fitting Residuals (Bq/mL×min^2^)	NoMC	1.43×10^−3^	9.54×10^−4^	2.16×10^−3^
	MC-IR	6.04×10^−3^	6.00×10^−3^	6.22×10^−3^
	MC-CR	1.81×10^−3^	1.77×10^−3^	1.81×10^−3^
	MC-CNN	3.33×10^−3^	2.92×10^−3^	3.11×10^−3^
	MC-CNN-C	7.47×10^−4^	7.82×10^−4^	7.93×10^−4^
	DeepMC	**6.29×10^−4^**	**6.06×10^−4^**	**6.24×10^−4^**
	Motion Free	6.20×10^−4^	6.20×10^−4^	6.20×10^−4^

**TABLE IV:** Summary of the MBFs an Fitting Residuals Measured on the 10 Cases With Real Motion for Different Motion Correction Methods. The Results Measured on Both the Whole LV Myocardium (Global) and the RCA ROIs Were Reported

	MBF (mL/min/g)		Fitting Residuals (Bq/mL×min^2^)	
	Global	RCA	Global	RCA
NoMC	1.64±0.86	1.90±1.17	4.5±2.4×10^−4^	9.6±6.6×10^−4^
MC-IR	3.23±1.96	3.81±2.11	2.6±4.7×10^−3^	2.3±1.9×10^−3^
MC-CR	1.91±1.04	1.66±1.00	1.1±1.0×10^−3^	1.1±0.8×10^−3^
MC-CNN	1.65±0.90	1.91±1.28	4.1±2.5×10^−4^	7.5±4.7×10^−4^
MC-CNN-C	1.64±0.86	1.78±1.01	5.1±5.2×10^−4^	9.5±7.6×10^−4^
DeepMC	1.61±0.84	1.83±1.08	4.4±2.8×10^−4^	7.8±4.7×10^−4^
