# Supplementary material for: Automatic Inter-Frame Patient Motion Correction for Dynamic Cardiac PET Using Deep Learning
Source: IEEE Trans Med Imaging. Author manuscript; Available in PMC 2021 Dec 14. (PMC8670362; doi:10.1109/TMI.2021.3082578)
Supplement: supp1-3082578 [file NIHMS1760792-supplement-supp1-3082578.docx]

**Supplementary Material**

1. **Details about the CNN for comparison**

Our proposed DeepMC was compared with another convolutional neural network (CNN). We chose a recent CNN architecture [1] that was designed for estimating rigid motion between two 3D image volumes. The network architecture that we used is shown in Figure. 1 (S). The network takes two 3D image frames (in two channels) as input and output the predicted translational motion displacement vector (*P*_x_, *P*_y_, *P*_z_), where *P*_x_, *P*_y_ and *P*_z_ are the predicted motion displacements in the left-right (x), anterior-posterior (y) and superior-inferior (z) directions. Note that we made a few changes compared with the original network structure [1]. The original network predicts both translational motion and rotational motion, therefore the last fully connected layer output a vector with 6 parameters. In our application, we only care about translational motion prediction, so we changed the last fully connected layer accordingly. We removed the batch normalization layers because we found they actually degraded the performance in our study. We also added dropout layers with rate 0.5 to the three fully connected layers in C8 to reduce overfitting.


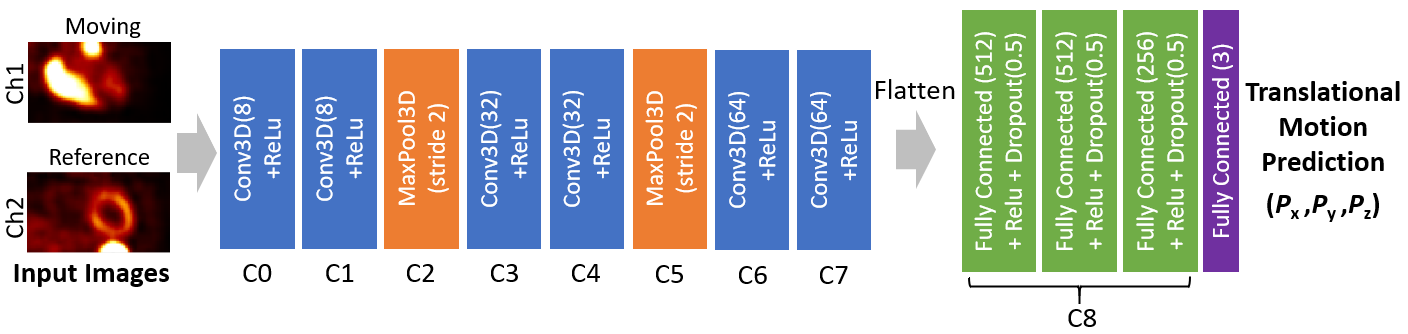


Figure. 1 (S). The CNN architecture used for comparison. All convolutional kernels have size 3×3×3. The input images in this figure show an example for MC-CNN, where the 2^nd^ channel is the reference frame (last frame in the sequence). For MC-CNN-C, the 2^nd^ channel would be the next frame of the moving frame (not shown here).

Similar to the conventional registration methods, we applied the CNN-based method in two strategies. In the first strategy, the CNN was trained and applied between each frame and the reference frame for motion estimation. In the second strategy, motion estimation was performed in a chain fashion, where the CNN was trained and applied between each frame and its next frame. We refer to these two methods as MC-CNN and MC-CNN-C (C stands for chain). For fair comparisons with DeepMC, we adopted the same convention to divide the image sequences into early and late frames for both MC-CNN and MC-CNN-C, so that each network can focus on a smaller problem for better performance. Since there is no need to append the reference frame to the end of early frames anymore (because temporal information is not used anymore), we included the first 14 frames as the early frames. The late frames included the last 13 frames, same as DeepMC.

Training MC-CNN and MC-CNN-C is slightly different from training DeepMC. Because each frame is processed individually, there is no need to simulate correlated motion (square motion and triangle motion). Therefore, only spike motion was randomly simulated for each frame (except for the first 3 frames and the reference frame), with the maximum motion shift limited to (4, 4, 4) voxels. The initial window shift was also applied randomly, as described in Section II.E. Each batch size in training included 4 motion replicates (from 4 patient scans), therefore the equivalent batch size was 4×14 = 56 for the early frames, and 4×13 = 52 for the late frames. A total of 100,000 motion replicate samples were randomly simulated based on the 65 patient scans described in section II.A, which is equivalent to 5.6 million samples for training the early frames, and 5.2 million samples for training the late frames. The mean square error (MSE) loss was used to update the network. The Adam optimizer with an initial learning rate of 0.001 was used, where the learning rate decayed by a factor of 0.999 for every 10 batches trained. The training took about 10 hours for either the early frames or the late frames.

1. **Training with a validation set**

Using a validation dataset can potentially help reduce overfitting and identify the best hyperparameter settings (in our case, the hyperparameter we want to optimize is whether or not we should apply each image pre-processing step as described in section II.D in the paper). Therefore, we further randomly divided the original training scan set (from 65) to 55 for training and 10 for validation, and used the validation error (MSE) to identify the best model for each group in the ablation study.

Note that in a common scenario with limited training data, the validation set is evaluated by the end of each training epoch (one complete pass through the training data) to determine the best model. However, in our case we can potentially have almost infinite training samples because we simulated motion replicates randomly. There is no ‘epoch’ in the conventional sense in our training settings. How to determine the size of the validation set is also a problem. Actually, in the modern big data era, the validation set and test set can take a much smaller percentage of the total data compared with the common 80%/10%/10% split for training/validation/testing. [2] suggests a 99.5%/0.25%/0.25% split, and we adopted this setting. In our study, we randomly simulated a large number (1 million) of motion replicates for training, so we used 1,000,000×0.25% = 2,500 motion replicates derived from the 10 patient scans as our validation set. The validation set, once generated for the first time, was kept fixed throughout the training and also across different groups (in the ablation study) for fair comparisons. During training, we evaluated on the validation set every 100 batches (this number was empirically chosen) trained, which results in 1,000,000/100/32 ≈ 312 times of evaluation on the validation set (32 is the batch size). We saved the model with the least validation error as our final model. The best models identified by the validation set were evaluated on the same evaluation set with 600 motion replicates.

As shown in Table I (S), the validation studies confirmed that the strategies presented in this paper are indeed optimal by incorporating all these image-preprocessing steps. However, splitting out a validation set from the training set will result in less training scan data (from 65 to 55), which, on the other hand, can also lead to reduced performance. We found that using the validation set resulted in larger motion estimation errors for most of the groups (except for No TN (Eval. w/o TN)), likely because of the reduction of the training scan set. This suggests that using a validation set can be helpful when there is an abundant training set, but might cause the opposite effect with a limited training set.

Table I (S). Ablation study results in terms of mean and max motion estimation errors across all the 600 evaluation samples. The results from models trained with and without a validation set are compared. IN: intensity normalization; TN: temporal normalization; IMC: iterative motion correction.

|  | Type | Mean Motion Err. (mm) | Max Motion Err. (mm) |
| --- | --- | --- | --- |
| Single Ch. Input | w/Val | 1.21±0.65 | 4.13±2.00 |
|  | w/o Val | 1.16±0.72 | 3.73±1.78 |
| No IN | w/Val | 0.97±0.50 | 3.89±2.05 |
|  | w/o Val | 0.96±0.48 | 3.70±1.82 |
| No TN (Eval. w/o TN) | w/Val | 2.06±1.07 | 14.78±8.71 |
|  | w/o Val | 2.50±1.94 | 22.27±16.36 |
| No TN (Eval. w/ TN) | w/Val | 1.14±0.64 | 4.28±2.13 |
|  | w/o Val | 1.03±0.52 | 3.85±1.62 |
| No IMC | w/Val | 1.17±0.72 | 4.93±2.34 |
|  | w/o Val | 0.99±0.63 | 3.92±1.98 |
| All applied | w/Val | 0.94±0.49 | 3.95±2.04 |
|  | w/o Val | 0.92±0.51 | 3.48±1.94 |

**Reference**

[1] S. S. M. Salehi, S. Khan, D. Erdogmus, and A. Gholipour, “Real-time deep pose estimation with geodesic loss for image-to-template rigid registration,” *IEEE transactions on medical imaging,* vol. 38, no. 2, pp. 470-481, 2018.

[2] C. L. Chowdhary, *Intelligent Systems: Advances in Biometric Systems, Soft Computing, Image Processing, and Data Analytics*: CRC Press, 2019.
